# 

**Published:** 1854-11

**Authors:** 


*New Books Received.*—Kölliker's Microscopical Anatomy, Skoda on Auscultation and Percussion, Meigs on Childbed Fever, Drake on the Fevers of the Interior Valley, and the Transactions of the Pennsylvania State Medical Society for 1854, are received and will have mention in our next.

*To Correspondents.*—The articles of Drs. Reynale and North, and the second article of Drs. Jameson and Avery, are obliged to lie over until the December number, when we hope to make a general delivery of the bag devoted to the favors of correspondents. H.

